# Evaluation of Aminoglycoside Dosing Regimens Adjusted for Renal Function and In Vitro Susceptibility Test Interpretive Criteria for Enterobacterales and *Pseudomonas aeruginosa*: Brief Summary

**DOI:** 10.1093/ofid/ofaf427

**Published:** 2025-11-26

**Authors:** Sujata M Bhavnani, Jeffrey P Hammel, Nikolas J Onufrak, Jason M Pogue, Ronald N Jones, Helio S Sader, George L Drusano, John S Bradley, David R Andes, William A Craig, Paul G Ambrose

**Affiliations:** Institute for Clinical Pharmacodynamics, Inc., Schenectady, New York, USA; Institute for Clinical Pharmacodynamics, Inc., Schenectady, New York, USA; Institute for Clinical Pharmacodynamics, Inc., Schenectady, New York, USA; Department of Clinical Pharmacy, College of Pharmacy, University of Michigan, Ann Arbor, Michigan, USA; JMI Laboratories/Element, North Liberty, Iowa, USA; JMI Laboratories/Element, North Liberty, Iowa, USA; Institute for Therapeutic Innovation, University of Florida, Orlando, Florida, USA; University of California at San Diego, San Diego, California, USA; Rady Children’s Hospital, San Diego, California, USA; Departments of Medicine and Medical Microbiology and Immunology, University of Wisconsin-Madison, Madison, Wisconsin, USA; Department of Medicine, University of Wisconsin-Madison School of Medicine and Public Health, Madison, Wisconsin, USA; Institute for Clinical Pharmacodynamics, Inc., Schenectady, New York, USA

**Keywords:** aminoglycosides, in vitro susceptibility test interpretive criteria, susceptibility breakpoints

## Abstract

The results of pharmacometric analyses for aminoglycosides were used to support recommendations for dosing regimens adjusted for renal function and a reassessment of the United States Committee on Antimicrobial Susceptibility Testing susceptibility test interpretive criteria for Enterobacterales and *Pseudomonas aeruginosa*. Susceptible and susceptible-based on therapeutic drug monitoring breakpoints are recommended for extended-interval amikacin, gentamicin, and tobramycin dosing regimens, thus representing adaptable treatable approaches.

## Teaser

A brief summary of the pharmacometric analyses for aminoglycosides used to support recommendations for dosing regimens adjusted for renal function and a reassessment of the USCAST STIC for Enterobacterales and Pseudomonas aeruginosa is provided. Susceptible and susceptible based on therapeutic drug monitoring breakpoints are recommended for extended interval amikacin, gentamicin, and tobramycin dosing regimens.

The aminoglycosides represent an older class of antimicrobial agents for which the pharmacokinetics–pharmacodynamics (PK-PD) for efficacy and safety were well characterized in the decades following their introduction. With this knowledge, aminoglycosides are now administered as larger doses with an extended interval to optimize efficacy and safety [1, 2]. However, recommendations for the administration of traditional or extended-interval aminoglycoside dosing regimens adjusted for renal function that match drug exposures for patients with normal renal function have been lacking. Also, given that in vitro susceptibility test interpretive criteria (STIC) for aminoglycosides were established more than 4 decades ago [3], updated recommendations were also needed.

Aminoglycoside STIC for Enterobacterales and *Pseudomonas aeruginosa* were reassessed in a comprehensive manner using a pharmacometric approach in 2019 by the United States Committee on Antimicrobial Susceptibility Testing (USCAST) [4, 5]. In 2023, another reassessment was undertaken by USCAST and these STIC were updated. Herein, we provide a brief summary of the recommendations for the administration of amikacin, gentamicin, and tobramycin dosing regimens adjusted for renal function and STIC criteria for these agents against Enterobacterales and *P. aeruginosa*. The results of the analyses that fully describe these recommendations are provided in the companion publication [6].

Table 1 shows the empiric aminoglycoside dosing recommendations based on renal function, measured by baseline creatinine clearance (mL/min), which are recommended for administration of traditional and extended-interval dosing of amikacin, gentamicin, and tobramycin. Therapeutic drug monitoring (TDM) should be performed, and using this information, dose modification can be made as needed to achieve target area under the plasma concentration-time curve (AUC) from time zero to 24 hours (AUC_0-24_) and minimum concentration (C_min_) measures associated with efficacy and safety, respectively [6, 9–12].

**Table 1.** Summary of Recommended Traditional and Extended-Interval Aminoglycoside Dosing Regimens^a,b^

Baseline Creatinine Clearance (mL/min)	Traditional and Extended-Interval Aminoglycoside Dosing Regimens					
	Amikacin		Gentamicin		Tobramycin	
	Traditional Dosing Regimen	Extended- Interval Dosing Regimen	Traditional Dosing Regimen	Extended-Interval Dosing Regimen	Traditional Dosing Regimen	Extended-Interval Dosing Regimen
>120 to ≤240	8 mg/kg q8h	20 mg/kg q24h	2 mg/kg q6h	7 mg/kg q24h	2 mg/kg q6h	7 mg/kg q24h
>90 to ≤120	8 mg/kg q12h	20 mg/kg q24h	2 mg/kg q8h	7 mg/kg q24h	2 mg/kg q8h	7 mg/kg q24h
>60 to ≤90	8 mg/kg q12h	20 mg/kg q36h	1.5 mg/kg q8h	7 mg/kg q36h	1.5 mg/kg q8h	7 mg/kg q36h
>45 to ≤60	8 mg/kg q24h	20 mg/kg q48h	1.5 mg/kg q12h	7 mg/kg q48h	1.5 mg/kg q12h	7 mg/kg q48h
>30 to ≤45	8 mg/kg q24h	20mg/kg q48h	1.5 mg/kg q12h	7 mg/kg q48h	1.5 mg/kg q12h	7 mg/kg q48h
≥16 to ≤30	8 mg/kg q48h	-	2 mg/kg q24h	-	2 mg/kg q24h	-

ABW, adjusted body weight; h, hours; IBW, ideal body weight; q, every; TBW, total body weight.

^a^Dosing regimens were based on TBW. Adjusted body weight was used in place of TBW if TBW was ≥25% higher than IBW. ABW was calculated for simulated patients as follows: ABW (kg) = IBW + 0.4 • (TBW - IBW) [7, 8].

^b^Based on providing the same exposure as normal renal function but safety for these dosing regimens has not been established.

Renally adjusted dosing regimen recommendations are needed for both traditional and extended-interval aminoglycoside administration. Traditional dosing recommendations for impaired renal function described in package inserts are vague and limited, stating that dose reductions by either using the normal dose with a prolonged interval or a reduced dose with a fixed interval should be employed. Extended-interval dosing is guided by dosing nomograms, the doses for which are intended to achieve high peak values with low or undetectable C_min_ values. Although maintaining low C_min_ values is important to the safe administration of aminoglycosides [9–12], the optimization of AUC values is associated with efficacy [6]. Thus, aminoglycoside dosing regimens adjusted for renal function using creatinine clearance (CLcr) were constructed with the goal of providing matching AUC and C_min_ values relative to those for patients with normal renal function (ie, CLcr >90 to ≤120 mL/min). Dosing regimens by CLcr group were selected using the following criteria: (1) ≤50% difference in median of the AUC_0-24_ values among simulated patients in each baseline CLcr group as compared to those with CLcr >90 to ≤120 mL/min; (2) ≤25% of simulated AUC_0-24_ values contained in the tails (5th, 95th percentiles) of the AUC_0-24_ distribution for simulated patients with CLcr >90 to ≤120 mL/min; and (3) ≤20% of simulated patients with C_min_ values >8 mg/L for amikacin and >2 mg/L for gentamicin or tobramycin in each CLcr group [9–12]. The recommended dosing regimens by CLcr group for amikacin, gentamicin, and tobramycin met these criteria.

STIC evaluations for antimicrobial agents are ideally informed from 3 data sources. These are the following: (1) in vitro surveillance data to evaluate the minimum inhibitory concentration (MIC) distributions that inform epidemiological cutoff (ECOFF) values; and the results of 2 endpoint-driven assessments, (2) the results of pharmacometric analyses to evaluate PK-PD target attainment by MIC value; and (3) evaluations of patient outcome by MIC value. Unfortunately, reports of patient outcome by MIC values to support STIC recommendations for the aminoglycosides were limited. Thus, the results of PK-PD target attainment analyses, based on the evaluation of nonclinical PK-PD targets for efficacy, were the primary endpoint-driven source of data informing USCAST STIC recommendations for aminoglycosides against Enterobacterales and *P. aeruginosa*.

With regard to the nonclinical endpoint for the PK-PD targets, it has been suggested that achieving PK-PD targets measured in serum or plasma that are associated with net bacterial stasis may be sufficient for efficacy against infections for which a drug concentrates at the effect site and/or those associated with lower bacterial burdens. For drugs that do not concentrate at the effect site and/or infections associated with higher bacterial burden such as pneumonia, endocarditis, or bacteremia, a 1-log_10_ CFU reduction from baseline may be a more appropriate nonclinical endpoint [13]. In the case of complicated urinary tract infections (cUTI), there may be an infection site with less drug penetration and where the pathogens are slowly growing for certain types (eg, enlarged prostate, foreign body) and thus, for which it is harder to effect a rapid kill. In such cases, a 1-log_10_ CFU reduction from baseline endpoint, which is the endpoint that has been the basis for regulatory assessments of dose and STIC for recently developed antimicrobial agents for infections arising from gram-negative infections, including cUTI [14, 15], may be more appropriate.

Given this information, and the intent to use a consistent metric for assessment across new and old antimicrobial agents, the USCAST STIC recommendations for aminoglycosides against Enterobacterales and *P. aeruginosa* were based on a 1-log_10_ CFU reduction from baseline endpoint, and as such, they are applicable to all systemic infections, including cUTI. The 2023 USCAST STIC recommendations for amikacin, gentamicin, and tobramycin for Enterobacterales and *P. aeruginosa* are summarized in Table 2. The susceptible breakpoints shown for each agent represent the highest MIC value at which the percent probabilities of PK-PD target attainment associated with this endpoint approached or were ≥90% with extended-interval dosing. The results of PK-PD target attainment analyses for extended-interval dosing were used for the STIC assessments as extended-interval dosing is guideline-recommended for aminoglycosides when treating patients with serious gram-negative infections [16–18]. Extended-interval dosing administration allows for drug exposures to be delivered once daily (or less frequently for patients with renal impairment), which relative to traditional interval dosing administration, optimizes efficacy given the concentration-dependent pattern of killing observed for this class [19] and safety by allowing for lower C_min_ values to be achieved.

**Table 2.** 2023 USCAST STIC Recommendations for Amikacin, Gentamicin, and Tobramycin Against Enterobacterales and *P. aeruginosa*

Organism/Antimicrobial	MIC Breakpoints in µg/mL^a^		
	Susceptible	Susceptible-TDM	Resistant
Enterobacterales			
Amikacin	≤2	4	≥8
Gentamicin	≤0.5	1	≥2
Tobramycin	≤0.5	1	≥2
*P. aeruginosa*			
Amikacin	≤2	4	≥8
Gentamicin	≤0.5	1	≥2
Tobramycin	≤0.5	1	≥2

AUC, area under the concentration-time curve; AUC:MIC ratio, ratio of the area under the concentration-time curve to minimum inhibitory concentration; MIC, minimum inhibitory concentration; PK-PD, pharmacokinetics–pharmacodynamics; S-TDM, susceptible breakpoint based on therapeutic drug monitoring; TDM, therapeutic drug monitoring.

^a^Based on the results of PK-PD target attainment analyses evaluating AUC:MIC ratio targets associated with a 1-log_10_ CFU reduction from baseline and the use of the extended-interval aminoglycoside dosing recommendations. Trough concentration monitoring is recommended for safety in all patients receiving aminoglycosides. An S-TDM category represents a susceptible breakpoint assuming TDM and dose adjustments to ensure that an AUC:MIC ratio target associated with a 1-log_10_ CFU reduction from baseline of approximately 84 is achieved.

The susceptibility category of “S-TDM,” which refers to susceptible based on therapeutic drug monitoring, was introduced and applied to all 3 aminoglycosides [6]. The premise for this category of breakpoints for each agent was that while administration of empiric dosing regimens shown in Table 1 cannot reliably achieve PK-PD targets at the MIC values representing the S-TDM breakpoints in 90% of patients, TDM and dose adjustment can be performed by clinicians caring for such patients to achieve targets at a given MIC value. Currently available safety data for gentamicin and tobramycin suggest that total-drug plasma AUC_0-24_ values necessary to achieve the ratio of the area under the concentration-time curve to MIC (AUC:MIC ratio) targets associated with a 1-log_10_ CFU reduction from baseline at the MIC values representing the S-TDM breakpoints can be safely administered [20, 21]. Because safety data were extrapolated to amikacin and robust evidence demonstrating safety at the exposures necessary for S-TDM breakpoints is lacking for this agent, caution is advised. Using this individualized approach, susceptible breakpoints that are 1 MIC dilution higher than the susceptible breakpoints shown in Table 2 can be used in clinical practice. However, the implementation of this susceptibility category in an institution would need to acknowledge and incorporate the fact that TDM is standard practice for aminoglycosides in the United States and in many other countries. For institutions for which TDM and TDM-associated dose adjustment is not possible, isolates with MIC values equal to the S-TDM breakpoint should be considered nonsusceptible.

As described previously, 1 data source used to inform STIC recommendations, and which is the sole data source considered for determination of ECOFF values, is MIC data for isolates collected through in vitro surveillance studies. USCAST aminoglycoside STIC for Enterobacterales and *P. aeruginosa* were considered in the context of MIC distributions for these pathogens. Although susceptible or S-TDM breakpoints that are high on the upper tail and do not bisect the MIC distribution were preferred to minimize antimicrobial susceptibility testing performance concerns, this characteristic was not the highest priority for USCAST STIC determinations, in contrast to those established by other organizations [22, 23]. It is the position of USCAST that STIC should be developed and implemented to best predict clinical outcomes, rather than to ensure reproducibility of the test with regard to categorical susceptibility. This position is the primary reason for the prioritization of PK-PD over in vitro MIC surveillance data to inform STIC. Nevertheless, susceptibility testing concerns with USCAST STIC recommendations are valid. As such, clinicians should be aware that the aminoglycoside susceptible and S-TDM breakpoints bisect the MIC distributions reported. Thus, because the typical normal variance within MIC testing can have a considerable impact on categorical testing interpretation, clinical decisions should also consider this variance.

The percentage of the total Enterobacterales and *P. aeruginosa* isolate collections and the drug-resistant subsets of Enterobacterales that are classified as susceptible and S-TDM based on the USCAST STIC are summarized in Table 3. The increase in percentage of isolates inhibited by each of the agents using the S-TDM instead of susceptible breakpoints ranged from 20.1% to 45.0% among collections including all isolates. Among the extended-spectrum β-lactamase subset, the increase in percentage of isolates inhibited ranged from 20.2% to 30.3%. Among the multidrug-resistant and carbapenem-resistant Enterobacterales (CRE) subsets, the increase in percentage of isolates inhibited ranged from 4.0% to 28.0%, the smallest of which were observed for tobramycin. Characterizing the percentage of isolates that are susceptible, especially when examined within an institution, provides a useful guide for the selection of empiric antimicrobial therapy.

**Table 3.** Percentage of Enterobacterales and *P. aeruginosa* Overall and for Drug-Resistant Subsets that are Classified as Aminoglycoside Susceptible and S-TDM Based on 2023 USCAST STIC

Pathogen	Agent	Isolate Collection	% Susceptible^a^	% S-TDM^a^
Enterobacterales	Amikacin	All isolates	72.7	94.4
		ESBL subset^b^	48.8	79.1
		MDR subset^c^	39.7	67.7
		CRE subset^d^	38.6	58.0
	Gentamicin	All isolates	68.1	88.2
		ESBL subset^b^	39.5	59.7
		MDR subset^c^	24.0	35.4
		CRE subset^d^	36.6	50.0
	Tobramycin	All isolates	55.4	82.5
		ESBL subset^b^	26.0	47.3
		MDR subset^c^	12.4	16.4
		CRE subset^d^	22.9	29.6
* P. aeruginosa*	Amikacin	All isolates	27.9	72.9
	Gentamicin	All isolates	7.60	30.9
	Tobramycin	All isolates	62.2	87.6

CRE, carbapenem-resistant Enterobacterales; ESBL, extended-spectrum β-lactamase; MDR, multidrug-resistant; S-TDM, susceptible breakpoint based on therapeutic drug monitoring; STIC, susceptibility test interpretive criteria; USCAST, United States Committee on Antimicrobial Susceptibility Testing.

^a^Based on the 2023 USCAST STIC recommendations for amikacin, gentamicin, and tobramycin for Enterobacterales and *P. aeruginosa* shown in Table 2.

^b^ESBL-phenotype isolates were defined as *Escherichia coli*, *Klebsiella pneumoniae, Klebsiella oxytoca, and Proteus mirabilis* displaying MIC values ≥2 mg/L for one of the following agents: aztreonam, ceftriaxone, and ceftazidime [22].

^c^MDR isolates were those that were nonsusceptible to ≥1 agents in ≥3 antimicrobial classes [24].

^d^CRE were isolates were those resistant to meropenem and/or imipenem when applying Clinical Laboratory and Standards Institute criteria [22].

Table 4 provides a comparison of the aminoglycoside STIC for Enterobacterales and *P. aeruginosa* established by USCAST [6], the Clinical Laboratory and Standards Institute (CLSI) [22], the United States Food and Drug Administration (FDA) [25], and the European Committee on Antimicrobial Susceptibility Testing [23]. Although the updated 2023 USCAST susceptible breakpoints are based on AUC:MIC ratio targets associated with a 1-log_10_ CFU reduction from baseline, CLSI and the recently updated FDA susceptible breakpoints are based on AUC:MIC ratio targets associated with net bacterial stasis with some exceptions [27] while those for European Committee on Antimicrobial Susceptibility Testing (EUCAST) are based on ECOFF values.

**Table 4.** Comparative STIC of USCAST, CLSI, the FDA, and the EUCAST

Pathogen/Agent	MIC Breakpoints in µg/mL by Organization			
	USCAST^a^	CLSI^b^/FDA^c^	EUCAST^d^
	Susceptible/Susceptible-TDM/Resistant	Susceptible/Resistant	
Enterobacterales			
Amikacin	≤2/4/ ≥8	≤4/ ≥16	≤8/ ≥16
Gentamicin	≤0.5/1/≥2	≤2/ ≥8	≤2/ ≥4
Tobramycin	≤0.5/1/≥2	≤2/ ≥8	≤2/ ≥4
*P. aeruginosa*			
Amikacin	≤2/4/ ≥8	≤16/ ≥64^e^	≤16/ ≥32
Gentamicin	≤0.5/1/≥2	…	…
Tobramycin	≤0.5/1/≥2	≤1/ ≥4	≤2/ ≥4

AUC, area under the concentration-time curve; AUC:MIC ratio, ratio of the area under the concentration-time curve to minimum inhibitory concentration; CLSI, The Clinical Laboratory and Standards Institute; ECOFF, epidemiological cutoff; EUCAST, European Committee on Antimicrobial Susceptibility Testing; MIC, minimum inhibitory concentration; PK-PD, pharmacokinetics–pharmacodynamics; S-TDM, susceptible breakpoint based on therapeutic drug monitoring; STIC, susceptibility test interpretive criteria; TDM, therapeutic drug monitoring; USCAST, United States Committee on Antimicrobial Susceptibility Testing; FDA, Food and Drug Administration; UTI, urinary tract infection.

^a^Based on the results of PK-PD target attainment analyses evaluating AUC:MIC ratio targets associated with a 1-log_10_ CFU reduction from baseline and the use of the extended-interval aminoglycoside dosing recommendations. Trough concentration monitoring is recommended for safety in all patients receiving aminoglycosides. An S-TDM category represents a susceptible breakpoint assuming TDM and dose adjustments to ensure that an AUC:MIC ratio target associated with a 1-log_10_ CFU reduction from baseline of approximately 84 is achieved.

^b^Based on Clinical Laboratory and Standards Institute M100-ED35 (2025) interpretive criteria. STIC are based on population distributions of various species, PK-PD target attainment analyses evaluating AUC:MIC ratio targets associated with net bacterial stasis and limited clinical data. Combination therapy for most indications other than UTI should be considered [22].

^c^Based on FDA interpretive criteria [25] for which the STIC based on CLSI M100-ED35 [22] is the recognized standard.

^d^Based on EUCAST 2025 breakpoint tables [23]. Represents STIC for systemic infections, which are based on ECOFF values. For such infections, aminoglycosides are recommended to be used in combination with other active therapy [26]. The same STIC are also recommended for infections originating from the urinary tract [23].

^e^Designated as urine STIC and reporting is only for isolates from the urinary tract [22].

The USCAST STIC recommendations for aminoglycosides represent a crucial step toward aligning modern dosing strategies with evolving clinical needs in an era of increasing antimicrobial resistance. By leveraging pharmacometric analyses, these recommendations are optimized for efficacy and safety through individualized renal function-based dosing and TDM. The introduction of the “S-TDM” category underscores the importance of adaptable treatment approaches, ensuring that aminoglycosides remain a valuable tool for treating patients with gram-negative infections. Moving forward, continued in vitro surveillance, clinical confirmation, and the integration of these findings into clinical practice will be essential to maximizing the utility of aminoglycosides while mitigating the risks associated with their use.

## Notes

***Acknowledgments.*** The authors would like to thank the FDA Division of Anti-Infectives for their prospective review of the analysis plan in 2017. The authors would also like to thank Drs. Thomas P. Lodise and Keith A. Rodvold for sharing their insights about the results of these analyses.

***Financial support.*** USCAST and the authors have received no funding for the studies and analyses undertaken.

***Patient consent statement and approval by local ethical committees.*** Patient consent was not applicable for the analyses undertaken. For nonclinical PK-PD studies, the data from which were used for the conduct of PK-PD target attainment analyses, animals were maintained in accordance with the guidelines of the Association for Assessment and Accreditation of Laboratory Animal Care International. All animal studies were approved by the Animal Research Committees of the William S. Middleton Memorial VA Hospital and the University of Wisconsin.

***Potential conflicts of interest.*** J. P. H, P. G. A., and S. M. B., as employees of the Institute for Clinical Pharmacodynamics (ICPD), were consultants for and received research funding from Achaogen Inc. S. M. B., as an employee of ICPD, was a consultant for and received research funding from Cipla Ltd. H. S. S., as an employee of JMI Laboratories/Element, was a consultant for and received research funding from Achaogen Inc. and Cipla Ltd. G. L. D. was an employee of the Institute for Therapeutic Innovation, University of Florida, a consultant for Achaogen Inc., and received research funding through an R01 grant R01AI090802 (Contact PI: Drusano) from the National Institute of Allergy and Infectious Diseases. All other authors report no potential conflicts.
